# Adherence to MIND Diet, Genetic Susceptibility, and Incident Dementia in Three US Cohorts

**DOI:** 10.3390/nu14132759

**Published:** 2022-07-03

**Authors:** Thanh Huyen T. Vu, Todd Beck, David A. Bennett, Julie A. Schneider, Kathleen M. Hayden, Aladdin H. Shadyab, Kumar B. Rajan, Martha Clare Morris, Marilyn C. Cornelis

**Affiliations:** 1 Department of Preventive Medicine, Northwestern University Feinberg School of Medicine, Chicago, IL 60611, USA; huyenvu@northwestern.edu; 2 Rush Institute for Healthy Aging, Chicago, IL 60612, USA; todd_beck@rush.edu (T.B.); kumar_rajan@rush.edu (K.B.R.); 3 Rush Alzheimer’s Disease Center, Chicago, IL 60612, USA; david_a_bennett@rush.edu (D.A.B.); julie_a_schneider@rush.edu (J.A.S.); 4 Department of Social Sciences and Health Policy, Wake Forest University School of Medicine, Winston-Salem, NC 27101, USA; khayden@wakehealth.edu; 5 Herbert Wertheim School of Public Health and Human Longevity Science, University of California, San Diego, CA 92093, USA; aladdinshadyab@health.ucsd.edu

**Keywords:** diet pattern, dementia, genotype, interaction

## Abstract

Adherence to Mediterranean-DASH Diet Intervention for Neurodegenerative Delay (MIND) may lower the risk of dementia by impacting immunity and cholesterol, which are pathways also implicated by genome-wide association studies of Alzheimer’s Dementia (AD). We examined whether adherence to the MIND diet could modify the association of genetic risk for AD with incident dementia. We used three ongoing US cohorts: Chicago Health and Aging Project (CHAP, *n* = 2449), Rush Memory and Aging Project (MAP, *n* = 725), and Women’s Health Initiative Memory Study (WHIMS, *n* = 5308). Diagnosis of dementia was based on clinical neurological examination and standardized criteria. Repeated measures of global cognitive function were available in MAP and CHAP. Self-reported adherence to MIND was estimated using food-frequency questionnaires. Global and pathway-specific genetic scores (GS) for AD were derived. Cox proportional hazard, logistic regression, and mixed models were used to examine associations of MIND, GS, and GS-MIND interactions with incident dementia and cognitive decline. Higher adherence to MIND and lower GS were associated with a lower risk of dementia in MAP and WHIMS and a slower rate of cognitive decline in MAP (*p* < 0.05). MIND or GS were not associated with incident dementia or cognitive decline in CHAP. No gene–diet interaction was replicated across cohorts. Genetic risk and MIND adherence are independently associated with dementia among older US men and women.

## 1. Introduction

The prevalence of dementia, particularly Alzheimer’s disease (AD), is expected to increase due to the progressive aging of the world population [1]. Pathological hallmarks of dementia accumulate decades before the onset of clinical symptoms; thus, preventive intervention strategies may help reduce the risk of developing the disease. Adherence to a Mediterranean-style dietary pattern may slow the rate of cognitive decline and decrease the risk of AD [2]. The Mediterranean-DASH Diet Intervention for Neurodegenerative Delay (MIND) is based on the most compelling evidence in the diet-dementia field [3]. MIND emphasizes consumption of green leafy vegetables, berries, nuts, beans, whole grains, seafood, poultry, olive oil, wine, and limited intake of animal and high-saturated-fat foods.

Genome-wide association studies (GWAS) of AD are enriched with single-nucleotide polymorphisms (SNPs) mapping to genes implicated in immune response and cholesterol metabolism [4]. Agnostic cross-phenotype genetic correlation and gene expression analyses corroborate these two pathways in AD development [5,6]. Adherence to a Mediterranean-style dietary pattern correlates with circulating markers of inflammation [7,8] and cholesterol metabolism [8,9]. Moreover, foods and nutrients emphasized by MIND demonstrate beneficial effects on the brain, potentially mediated by inflammation or cholesterol pathways [10,11,12,13]. MIND associations with cognitive decline are modest. However, no study has examined the interaction between adherence to MIND and genetic susceptibility to dementia risk. When combined with individual genetic information concerning AD susceptibility, this interaction may reveal population subgroups who might benefit from adherence to this diet.

The current study examines whether genetic differences in AD predisposition modify the association between MIND adherence and incident dementia. We hypothesized that participants with greater genetically inferred AD risk, particularly in pathways concerning immune response and cholesterol metabolism, would benefit the most when adhering to MIND compared to participants with lower susceptibility profiles.

## 2. Methods

### 2.1. Study Populations

The current analysis included three US cohorts: Chicago Health and Aging Project (CHAP), Rush Memory and Aging Project (MAP), and Women’s Health Initiative Memory Study (WHIMS). CHAP is a biracial cohort with 60% African Americans that began in 1993 with a census of individuals ages ≥ 65 years sampled from four adjacent neighborhoods in South Side, Chicago, Illinois [14]. Of those identified, 6158 persons (79%) participated in a home interview. Additional people enrolled as they turned 65 years old, for 10,802 participants enrolled through 2012 [15]. The current analysis was limited to CHAP participants with genetic and diet data. MAP began in 1997 and is an open cohort of residents from individual homes, retirement communities, and senior public housing units in the Chicago, Illinois area [16]. Participants are free of dementia at enrollment and agree to annual clinical evaluations and organ donation at death. Participants were invited to complete food frequency questionnaires (FFQs) during their annual clinical evaluations from 2004 to 2013. A total of 1306 participants were either currently enrolled or newly enrolled during this period (‘baseline’ for the current analysis). WHIMS is an ancillary study to the WHI hormone therapy trials [17,18,19,20,21,22]. From 1995–1999, 7479 women ≥ 65 years of age who were free of dementia were recruited at 39 US clinical centers and completed annual clinic-based cognitive assessments [17,19,22]. In 2008, women actively enrolled in WHIMS were invited to continue follow-up in the WHIMS-Epidemiology of Cognitive Health Outcomes (WHIMS-ECHO) study, which replaced clinic-based assessments with telephone-based assessments. For the current WHIMS analysis, we considered only women of European ancestry (~89% of cohort) with genetic data. An Institutional Review Board at each academic center overseeing each cohort approved the study. All cohort participants gave written informed consent. MAP participants signed a repository consent allowing their data to be shared.

### 2.2. Diet Assessment and MIND Adherence Score

CHAP, MAP, and WHIMS participants completed validated semi-quantitative FFQs that estimated daily food/beverage and nutrient intake during the previous year (MAP, CHAP) or 3-month period (WHIMS). We used data from the first administered FFQ for the current analysis. For MAP and WHIMS, the first FFQs were completed at study entry (baseline). CHAP participants completed the first FFQ at a median of 0.5 years from baseline. Responses from about 15 food-items were used to derive a MIND adherence score as described previously [23] and detailed in Supplementary Table S1. Each food-item corresponds to 1 point if adhered to, with a total max score of 15. Half points for partial adherence are also pre-specified. Study-specific modifications were made to account for differences in FFQs.

### 2.3. Cognitive Assessments

All CHAP and MAP participants completed a battery of cognitive tests administered by trained research staff/nurses during in-home assessments (CHAP) or psychologists/nurses during clinical visits (MAP) (Supplementary Table S2). For each participant, a global measure of cognition was computed for each clinical visit by standardizing raw scores for each test using the mean and standard deviation from the baseline population scores and the standardized scores averaged. WHIMS participants were screened annually with the Modified Mini-Mental State Examination (3MSE) [24], which generates a global score ranging from 0 (low) to 100 (high cognitive function). If a participant scored at or below the cut point (80 points if ≤8 years of formal education and 88 points if ≥9 years of formal education), she progressed to further in-person cognitive testing (Supplementary Table S2). For WHIMS-ECHO (2008–present), 3MSE was replaced with the Modified Telephone Interview for Cognitive Status (TICSm), which generates a score ranging from 0 (low) to 50 (high cognitive function) [25]. Scores < 30 trigger administration of the Dementia Questionnaire [26] to a proxy informant.

### 2.4. Dementia Outcomes

In CHAP, clinical evaluations for Alzheimer’s disease and dementia diagnosis were performed in a stratified random sample of the study population [14,15,27,28]. Briefly, from the surviving cohort determined to be free of dementia at the previous cycle, sampling for clinical evaluation of incident dementia in cycles 2–6 was stratified by age, race, sex, and change in cognitive function from the previous home interview, with participants selected randomly from all strata (4021 evaluations on 2794 participants between 1994 and 2012). Diagnosis of Alzheimer’s dementia was based on criteria of the joint working group of the National Institute of Neurological and Communicative Disorders and Stroke and the Alzheimer’s Disease and Related Disorders Association [29], which require a history of cognitive decline with impairment in memory and at least one other cognitive domain. In MAP, a clinical diagnosis of (all-cause) dementia and Alzheimer’s dementia was determined at each annual evaluation using the same methodology as in CHAP [30]. In WHIMS, those undergoing cognitive test assessments (after 3MSE screen) were evaluated by a local physician. Using a standardized protocol provided by the WHIMS clinical center, the physician performed a clinical neuropsychiatric evaluation with all available data and classified the participant as having no dementia, mild cognitive impairment (MCI), or probable dementia (herein referred to as all-cause dementia), based on criteria from the Diagnostic and Statistical Manual of Mental Disorders (4th ed.) [17]. Clinical and test data were then sent to the WHIMS Clinical Coordinating Center for central adjudication of final diagnosis by a clinical panel with expertise in dementia diagnosis. Central adjudication of final diagnosis was similar in WHIMS-ECHO.

### 2.5. Genetic Data

CHAP participants self-reporting as white (CHAP-White) or black (CHAP-Black) were genotyped using the Affymetrix and Illumina Human Omni1Quad platforms. MAP analyses were restricted to MAP participants self-reporting as non-Hispanic white. They were genotyped on the Illumina Global Screening Array, Illumina Human1M, and Affymetrix 6.0 platforms as described in detail previously [31,32]. All WHIMS participants who provided consent had GWAS data in either the “WHI WHIMS+ GWAS” (phs000675.v3.p3, HumanOmniExpressExome-8 v1.0 platform) or “WHI GARNET” (phs000315.v7.p3, Illumina Omni-Quad platform) studies. A recent effort combined these and four other WHI GWAS data sets to generate the “WHI Harmonized and Imputed GWAS Data” (phs000746.v2.p3); this was a resource used for the current study but only for the genetically inferred and European ancestry WHIMS subset. CHAP, MAP and WHIMS were imputed using at least the 1000G v3 ALL reference panel. APOE genotyping was performed using Sequenom MassARRY for CHAP participants and sequencing for MAP participants. For WHIMS, APOE carriers(ε4+) and non-carriers(ε4-) were defined using SNPs rs7412 and rs429358.

### 2.6. Other Covariates

Each cohort collected several medical, physical, demographic, and lifestyle data at baseline and throughout follow-up via self-administered questionnaires or structured interviews during clinical visits. Data on education, socioeconomic status, medical history, depressive symptoms, smoking status, total energy intake (derived from FFQ), body mass index (BMI, kg/m^2^), and physical activity were collected and detailed in Supplementary Table S3. The late-life cognitive activity was also measured in CHAP and MAP. For dementia analysis, we considered covariates measured at baseline or closest to the time of the first FFQ. Time-varying covariates were considered in cognitive decline analyses of the MAP cohort.

### 2.7. Final Analytical Samples

The initial sample size of participants with available baseline diet and outcome data was 6856, 1054 and 6851 for CHAP, MAP and WHIMS, respectively. Corresponding sample sizes after excluding participants without genetic data, less than one follow-up, and prevalent dementia cases (MAP) were 2449 (max for cognitive decline), 725 and 5308 (see Supplementary Table S4 for sample exclusion details).

### 2.8. SNP-Selection and Calculation of Genetic Susceptibility Scores (GS)

A genetic susceptibility score for AD (GS_AD_) was computed using 25 SNPs with MAF > 1% and reaching GW significance level (*p* < 5 × 10^−8^) in GWAS of AD (Supplementary Table S5). GS_AD_ uses the sum of the products of SNP risk alleles and their corresponding weights as GS_AD_ = Σ_i_^n^ log(OR_ij_) × G_ij_ for the ith individual, where log(OR_ij_) = the log of the OR for the jth SNP, G_ij_ = the number of risk alleles (0, 1, or 2) for the jth SNP and *n* = 25 candidate SNPs. The score is rescaled according to the number of risk alleles (#SNPs × 2) to facilitate interpretation. Higher GS_AD_ scores correspond to elevated AD risk. GS_AD-I_ and GS_AD-C_ are subset scores of GS_AD_ and include SNPs mapping to, respectively, the immune response (10 SNPs) and cholesterol metabolism (4 SNPs) pathways (Supplementary Table S5). *APOE* was not included in the GSs because of its large effect size but was examined separately.

### 2.9. Statistical Analysis

All statistical analyses were performed using the SAS v9.4 statistical package (SAS Institute, Cary, NC, USA) and separately for each cohort and ancestry (CHAP only). We first examined the main effects of MIND and GS on dementia risk and cognitive decline before proceeding to formal tests of GS × MIND interactions. Main effects of MIND (MAP [23], WHIMS [33]), GS_AD_ (MAP [34]), and *APOE* (MAP [16], WHIMS [35], CHAP [36]) have been published previously but using different statistical models, larger sample sizes and/or different GS. For MAP and WHIMS, Cox proportional hazard regression models examined the association between MIND adherence (or GS), expressed in tertiles, and incident dementia. To test for linear trends, MIND (or GS) was entered into the model as a continuous term. MAP participants were considered at risk for dementia from 2004 and were followed up until the date of first clinical diagnosis or last clinical exam, whichever came first. WHIMS participants were considered at risk for dementia from 1995 and were followed up until the date of first probable dementia classification, death, loss to follow-up, or 1 January 2019 (the last date of dementia reported), whichever came first. For each cohort, the proportionality of hazards assumption was assessed using time-dependent explanatory variables and Schoenfeld residuals techniques [37] and was satisfied. For CHAP-White and CHAP-Black, a sample-weight-adjusted logistic regression model was used to estimate the odds ratio (OR) for developing dementia, as described previously [38]. A delete-a-group jackknife method was used to estimate the standard error of risk estimates and account for the stratified random sampling for the participants chosen for the clinical diagnosis from the population sample.

A “Basic” model adjusted for age, sex (MAP and CHAP), region (WHIMS), randomization status (WHIMS) and, in genetic analysis only, genotyping platform (MAP and WHIMS) and 10 ancestry principal components (PCs, WHIMS and CHAP). To this model, we added covariates capturing measures of cognitive reserve, cardiovascular/metabolic health and lifestyle, which may confound the association between MIND and cognitive impairment (‘Full” model). Model covariates differed by cohort and are detailed in Supplementary Table S3. Missing data were present in some covariates (up to 5%) and were modeled as indicator variables (Supplementary Table S3).

For cognitive decline analysis in MAP and CHAP, linear-mixed models with random intercept and time (slope) estimated mean differences in annual rates of cognitive decline by tertile of MIND (or GS). Models included covariates described above as well as a variable for time, and multiplicative terms between time and each model covariate. The latter provides the covariate estimated effect on cognitive change. WHIMS was not included for cognitive decline analysis since too few women had repeated 3MSE measures.

Gene × MIND interactions for incident dementia and cognitive decline were tested by including the gene (GS_AD_, GS_AD-C_, GS_AD-I,_ or APOE ε4 carrier status), MIND score, and their cross-product term in basic model regressions. Corresponding terms in linear-mixed models include gene × time, MIND × time, and gene × MIND × time. Statistical significance was defined as *p* < 0.05 and consistency across cohorts.

## 3. Results

### 3.1. Baseline Characteristics of Study Populations

At baseline, MAP participants were older and more likely to have a history of hypertension, heart disease, and stroke compared to participants in the other cohorts. CHAP-Black participants were more likely to present with moderate cognitive impairment, diabetes, and depression. CHAP-Black participants had slightly lower GS_AD_, GS_AD-I,_ and GS_AD-C_ but a higher proportion of APOE ε4 carriers compared to the other cohorts. Figure S1 presents cohort distributions of the MIND adherence score. Mean MIND adherence scores were 7.9, 6.7, 7.4 and 7.1 for MAP, WHIMS, CHAP-White and CHAP-Black cohorts, respectively. Across cohorts, participants with higher adherence to MIND were more likely to be female, non-smokers, highly educated and have better cognitive ability, while less likely to have a history of stroke or depression (Table 1). MIND adherence also correlated with age, BMI, and other medical conditions, but these correlations differed by cohort.

### 3.2. Main Effects of MIND Adherence and Genetic Factors on Incident Dementia and Cognitive Decline

During follow-up, 222, 951, 67, and 109 participants developed dementia in MAP, WHIMS, CHAP-White and CHAP-Black, respectively. Mean age of onset was 90 (MAP), 86 (WHIMS), 79 (CHAP-White), and 77 (CHAP-Black) years. MAP and WHIMS participants in the highest tertile of MIND adherence had a significantly lower risk of all-cause dementia compared to those in the lowest tertile after full model adjustment (*p* < 0.02, Table 2). MAP participants in the highest tertile of MIND adherence also had a significantly lower risk of Alzheimer’s dementia [HR (95%CI): 0.62 (0.42, 0.92)] and a lower rate of cognitive decline (Table 3) compared to those in the lowest tertile. Adherence to MIND was not associated with incident all-cause dementia (Table 2), Alzheimer’s dementia (*p* > 0.05, not shown) or cognitive decline (Table 3) in CHAP. In post hoc analysis, significant sex × MIND interactions for all-cause (*p* = 0.03) and Alzheimer’s (*p* = 0.04) dementia were observed in CHAP-Black. MIND was associated with a lower risk of all-cause dementia in Black men (OR = 0.65, *p* = 0.004) but not in Black women (OR = 1.22, *p* = 0.27). A similar pattern was observed for Alzheimer’s dementia. Results were similar when excluding prevalent diabetes (data not shown).

GS_AD_ and GS_AD-I_ were positively associated with incident dementia in MAP and WHIMS cohorts but not in CHAP cohorts (Table 4). Similar results were observed for Alzheimer’s dementia (Supplementary Table S6). GS_AD_ and GS_AD-I_ were positively associated with the rate of cognitive decline in MAP but not CHAP cohorts (Supplementary Table S7). *APOE* ε4 carriage was a significant risk factor for all-cause dementia in MAP [HR (95%CI): 1.99 (1.49, 2.66)] and WHIMS [HR (95%CI): 2.08 (1.89, 2.30)] but not in CHAP-White [HR (95%CI): 1.80 (0.76, 4.29)] or CHAP-Black [HR (95%CI): 1.20 (0.46, 3.15)] cohorts. *APOE* ε4 carriers also presented with significantly greater rates of cognitive decline in MAP, CHAP-White and CHAP-Black (all *p* < 0.0001).

### 3.3. Gene × MIND Interactions for Incident Dementia and Cognitive Decline

**Figure 1:**
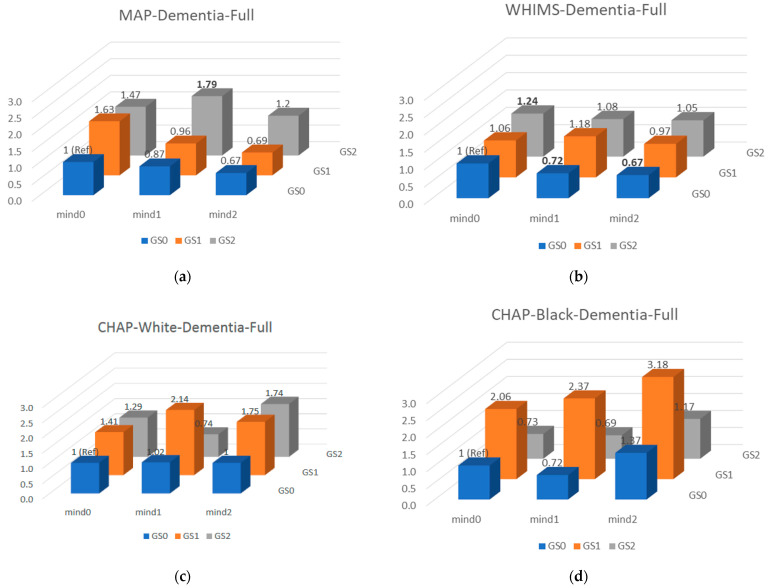
Joint analysis of GS_AD_×MIND and all-cause dementia. Results from Cox proportional hazard (HR, MAP (**a**)/WHIMS (**b**)) or logistic (OR, CHAP (**c**,**d**)) regression models adjusted for age, sex (MAP, CHAP only), study center and randomization status (WHIMS only), education, income, global cognition score, late-life cognitive activity (MAP, CHAP only), history of diabetes, hypertension, stroke and heart disease; smoking, calorie intake, BMI, depressive symptoms, physical activity, genotype platform (MAP) or PCs (CHAP, WHIMS). Mind(GS)1, Mind(GS)2, Mind(GS)3 indicate tertile 1, tertile 2 and tertile 3 MIND adherence (GS) scores, respectively. Significant HR/ORs are bold-faced.

Joint analysis for GS_AD_×MIND and all-cause dementia are presented in Figure 1. Only cohort-specific gene × MIND interactions were observed (Table 5); no interactions replicated across cohorts. Post hoc fixed effects meta-analyses supported interactions but were largely driven by WHIMS and difficult to interpret. For example, *APOE* × MIND interactions for all-cause dementia and Alzheimer’s dementia are in opposite directions. Results were similar when excluding prevalent diabetes (data not shown).

## 4. Discussion

Adherence to MIND may lower the risk of AD by impacting immunity and cholesterol; pathways also implicated by GWAS of AD [4,7,8,9]. Since diet and genetic factors targeting the same pathway might act synergistically when combined, we hypothesized that participants with greater genetic AD susceptibility, particularly in pathways concerning immune response and cholesterol metabolism, would benefit the most when adhering to MIND compared to participants with lower susceptibility profiles. Our findings, based on three aging cohorts, do not support our hypothesis and suggest independent roles of MIND and genetic susceptibility on dementia development.

To our knowledge, this is the first study to examine the interaction between MIND adherence and a comprehensive AD GS. Samuelsson et al. [39] recently examined posterior-derived dietary patterns, *p*-value threshold-based polygenic AD risk scores, and *APOE* in 602 elderly of the Gothenburg H70 Birth Cohort Studies in Sweden. None of the polygenic risk scores or dietary patterns alone were significantly associated with dementia risk. However, *APOE* ε4 carriers with a higher adherence to a “western dietary pattern” presented with an increased risk of dementia compared to those with lower adherence to this dietary pattern; no association between diet and risk was observed among non-carriers [39]. Hossain et al. [40] examined diet × GS interactions on performance changes in 11 cognitive tests in up to 230 African Americans from the Healthy Aging in Neighborhoods of Diversity across the Life Span Cohort study. A diet quality score was derived from PC analyses of three dietary patterns including Health Eating Index 2010, DASH, and mean adequacy ratio. The AD GS included 2 SNPs in *APOE* and 10 others selected from the literature but not confirmed in GWAS. Only sex-specific main effects of the diet score and GS were observed for different cognitive tests. Moreover, improvements in diet quality over time were associated with a slower rate of memory decline only among those with a higher AD GS. Neither study included an independent replication cohort and the diets and GS considered were much different from those of the current study.

To protect against false-positive interactions, we included three independent aging cohorts to allow for replication. Indeed, had we included only one of these studies, we would have reported a non-replicable interaction. However, efforts to replicate interactions face practical and conceptual challenges [41]. For example, the availability of large genotyped and well-phenotyped aging cohorts to address our study objective was limited. Our efforts to harmonize analyses came at the cost of data quality, which tends to correlate with sample size. MAP and CHAP may not have been sufficiently powered to detect interactions identified in WHIMS, especially if they were specific to women. The less precise methods of case-ascertainment in CHAP and WHIMS compared to MAP may have hindered the replication of interactions observed in MAP. Nevertheless, regardless of statistical significance, similar patterns of interactions were also not observed across cohorts. Thus, had a true diet × gene interaction been overlooked, it would likely have limited generalizability.

While the main effects of MIND and GS on cognitive impairment have been published previously and in independent cohorts, re-examining these in the current study partly informed the power of the study to test interactions, specifically, fan-shaped interactions [41]. Cross-over MIND × GS interactions are conceptually unlikely and would require even more power to replicate. The current study reports the first analyses of MIND in CHAP. MIND was not associated with incident dementia or cognitive decline in either CHAP sample. In a larger sample of CHAP, higher adherence to the Mediterranean-type dietary pattern was associated with a slower rate of cognitive decline [42]. MIND differs from the Mediterranean-type dietary pattern by allocating separate categories for green leafy vegetables and berries, and a category for cakes/pastries and fast fried foods. MIND also does not include potatoes or other fruit and fish is prescribed less frequently than the Mediterranean-type dietary pattern. The main effects of MIND on cognitive impairment have not been observed universally [43]. In the Rotterdam Study, MIND was associated with a lower risk of all-cause dementia over the first seven years of follow-up, but associations disappeared with longer follow-up, consistent with the notion of reverse causation [44]. This does not likely explain the lack of an association between MIND and dementia in CHAP, since this cohort had a shorter follow-up than MAP and WHIMS. The distribution of MIND scores across cohorts was not perfectly aligned and thus our tertile-based analyses introduced sample-specific cut-points. However, cut-points for WHIMS and CHAP were very similar and tertile tests were complemented by continuous linear tests, which consider the full distribution of scores. Limited sample power (cases and total sample size) or other characteristics unique to CHAP may explain the lack of an association between MIND and incident dementia in this cohort.

The main effects of MIND, GS and *APOE* on dementia risk were generally confirmed in MAP and WHIMS but not CHAP. The GS was based on loci identified in GWAS of populations of European-ancestry and thus may not be appropriate for predicting dementia in other ancestries. More genetic discovery studies involving other ancestries are needed. However, the inability to replicate genetic findings in either CHAP-Black or CHAP-White suggest factors beyond ancestry are at play. Sample size, physical location, smoking and other demographic characteristics unlikely explain discrepancies since these were shared across cohorts. We also considered the higher prevalence of diabetes in CHAP compared to MAP and WHIMS by excluding prevalent diabetes from all cohorts; discrepancies remained. Interestingly, *APOE* was strongly associated with cognitive decline in both CHAP samples. It is also possible that the stratified sampling approach to case-ascertainment induced some form of bias not observed in repeated measures of cognitive function.

Studies reporting significant interactions between lifestyle factors and *APOE* have been conflicting. Lifestyle modification of risk is more pronounced among *APOE* ε4 carriers in some studies [45,46,47,48] while more pronounced among non-carriers in other studies [49,50,51,52,53]. In the large population-based UK biobank, a healthy lifestyle (defined by smoking status, physical activity, diet and alcohol intake) was associated with lower risk of dementia among participants with both a high and low AD GS [54]. The notion that individuals may modify their risk of dementia through a healthy lifestyle regardless of genetic susceptibility has important public health implications. Inconsistencies in the literature emphasize the need for more pooled or multi-cohort analyses of lifestyle × gene interactions for dementia.

The use of three well-characterized community- or population-based aging cohorts with detailed neurological assessments and standardized dementia ascertainment methods are key strengths of the current study. The complementary genetic, covariate and diet data also enabled a harmonized approach to the analysis. Nevertheless, several limitations should be acknowledged in addition to those discussed above. Individuals may be free of clinical dementia symptoms but may already have biomarkers of the disease. The time of diet collection in these cohorts may not have adequately captured earlier diet behaviors which might have a greater impact on disease pathways (i.e., cholesterol or immunity) to influence outcomes. Unlike genetic factors, diet and other modifiable behaviors are not randomly assigned at birth and thus unmeasured confounding and reverse causation remain possible.

## 5. Conclusions

Among older US men and women, genetics and adherence to MIND were independently associated with dementia risk. Our study currently supports advocating MIND more generally as opposed to targeting specific subgroups defined by genetics.

## Figures and Tables

**Table 1:** Baseline characteristics of participants by tertiles of MIND score ^1^.

	MAP			WHIMS			CHAP-White			CHAP-Black		
Characteristic	T1	T2	T3	T1	T2	T3	T1	T2	T3	T1	T2	T3
	3.5–7.0	7.5–8.5	9.0–13.0	2–5.5	6–7	7.5–12	2–6	6.5–7.5	8–14	2–6	6.5–7.5	8–12.5
	n = 270	n = 233	n = 222	n= 1485	n = 1917	n = 1906	n = 255	n = 300	n = 391	n = 475	n = 513	n = 515
Age, years	82.3 ± 7.2	82.5 ± 6.5	80.3 ± 6.8	69.8 ± 3.8	70.2 ± 3.85	70.3 ± 3.8	74.0 ± 6.3	74.2 ± 6.3	72.2 ± 5.7	71.7 ± 4.6	71.9 ± 4.5	71.1 ± 4.1
Male, n (%)	74 (27)	60 (26)	52 (23)	0 (0)	0 (0)	0 (0)	123 (48)	105 (35)	130 (33)	218 (46)	176 (34)	162 (31)
Baseline cognitive function, Z-score	−0.05 ± 0.57	0.13 ± 0.50	0.30 ± 0.45	−0.08 ± 1.01	0.01 ± 1.01	0.04 ± 0.99	0.61 ± 0.53	0.69 ± 0.52	0.81 ± 0.40	0.23 ± 0.58	0.31 ± 0.60	0.40 ± 0.56
^2^ Mild cognitive impairment, n (%)	80 (30)	56 (24)	33 (15)	0 (0)	0 (0)	0 (0)	40 (16)	41 (21)	34 (12)	79 (27)	89 (31)	78 (39)
Education												
College or University degree, n (%)	n/a	n/a	n/a	318 (21)	585 (31)	755 (40)	n/a	n/a	n/a	n/a	n/a	n/a
Years of education	14.3 ± 3.1	15.1 ± 2.8	15.5 ± 2.7	n/a	n/a	n/a	14.1 ± 3.0	14.6 ± 3.2	15.3 ± 3.3	11.3 ± 3.2	12.0 ± 3.2	12.7 ± 3.2
Hypertension, n (%)	202 (75)	168 (72)	152 (68)	562 (38)	756 (39)	684 (36)	101 (40)	131 (44)	167 (43)	292 (62)	324 (63)	346 (67)
Diabetes, n (%)	46 (17)	27 (12)	25 (11)	105 (7)	136 (7)	136 (7)	31 (12)	28 (9)	39 (10)	102 (21)	129 (25)	123 (24)
Stroke, n (%)	38 (14)	17 (7)	22 (10)	23 (2)	32 (2)	19 (1)	19 (7)	21 (7)	18 (5)	41 (9)	41 (8)	35 (7)
Heart disease, n (%)	55 (20)	31 (13)	38 (17)	254 (17)	310 (16)	324 (17)	20 (8)	38 (13)	55 (14)	56 (12)	66 (13)	50 (10)
Current smoker, n (%)	9 (3)	2 (1)	2 (1)	127 (9)	123 (6)	76 (4)	34 (13)	25 (8)	25 (6)	70 (15)	65 (13)	57 (11)
BMI, kg/m^2^	27.5 ± 5.2	27.3 ± 5.0	26.6 ± 5.6	29.2 ± 5.7	28.5 ± 5.8	27.7 ± 5.3	26.7 ± 5.3	27.1 ± 4.9	27.2 ± 4.9	28.9 ± 6.2	29.4 ± 6.3	29.2 ± 5.6
^3^ Depression/symptoms, n (%)	18 (7)	8 (3)	3 (1)	138 (9)	132 (7)	109 (6)	20 (8)	8 (3)	14 (4)	62 (13)	58 (11)	47 (9)
MIND score	6.1 ± 0.8	8.0 ± 0.4	9.9 ± 0.9	4.8 ± 0.7	6.5 ± 0.4	8.3 ± 0.8	5.2 ± 0.9	7.0 ± 0.4	9.1 ± 1.1	5.3 ± 0.7	7.0 ± 0.4	8.8 ± 0.9
Apoe ε4 carriers, n (%)	57 (21)	44 (19)	47 (21)	187 (13)	226 (12)	262 (14)	63 (25)	69 (23)	109 (28)	173 (36)	174 (34)	206 (40)
GS_AD_	26.6 ± 3.0	26.8 ± 2.9	26.7 ± 2.8	26.9 ± 3.0	26.8 ± 3.1	26.9 ± 3.0	26.5 ± 3.0	27.1 ± 3.0	27.0 ± 3.0	24.4 ± 2.6	24.5 ± 2.8	24.4 ± 2.6
GS_AD-I_	9.6 ± 2.1	9.7 ± 2.0	9.5 ± 1.9	9.7 ± 2.1	9.6 ± 2.2	9.6 ± 2.1	9.6 ± 2.2	9.8 ± 2.1	9.6 ± 2.2	9.6 ± 1.6	9.4 ± 1.6	9.4 ± 1.7
GS_AD-C_	5.5 ± 0.9	5.6 ± 0.9	5.5 ± 1.0	5.52 ± 0.95	5.49 ± 0.96	5.49 ± 0.97	5.5 ± 1.0	5.5 ± 0.9	5.6 ± 0.9	5.0 ± 1.0	5.0 ± 1.0	5.0 ± 0.9

^1^ Values are mean ± SD or *n* (%); n/a: information not available or applicable. ^2^ Weighted % for CHAP. ^3^ CESD-10 ≥ 5 (MAP, CHAP); shorter-form CESD (Burnam scoring) ≥ 0.06 (WHIMS).

**Table 2:** MIND score and risk of all-cause dementia.

Model	MAP		WHIMS		CHAP-White		CHAP-Black	
	HR (95% CI)	*p*	HR (95% CI)	*p*	OR (95% CI)	*p*	OR (95% CI)	*p*
Basic ^1^								
MIND T1	Ref.		Ref.		Ref.		Ref.	
MIND T2	0.70 (0.52, 0.94)	0.02	0.88 (0.80, 0.97)	0.008	0.54 (0.21, 1.43)	0.22	0.82 (0.35, 1.95)	0.66
MIND T3	0.43 (0.30, 0.61)	<0.0001	0.78 (0.70, 0.86)	<0.0001	0.49 (0.18, 1.33)	0.16	0.97 (0.32, 2.87)	0.95
Trend	0.84 (0.77, 0.91)	<0.0001	0.93 (0.91, 0.96)	<0.0001	0.81 (0.61, 1.09)	0.17	0.99 (0.73, 1.34)	0.96
Full ^2^								
MIND T1	Ref.		Ref.		Ref.		Ref.	
MIND T2	0.85 (0.62, 1.16)	0.31	0.87 (0.79, 0.97)	0.008	0.87 (0.30, 2.54)	0.80	0.86 (0.36, 2.05)	0.74
MIND T3	0.63 (0.42, 0.92)	0.02	0.80 (0.72, 0.89)	<0.0001	1.23 (0.47, 3.18)	0.68	1.48 (0.51, 4.27)	0.47
Trend	0.91 (0.83, 1.00)	0.06	0.95 (0.92, 0.97)	<0.0001	1.00 (0.81, 1.25)	0.97	1.08 (0.79, 1.48)	0.61

^1^ Results from Cox proportional hazard (HR) or logistic (OR) regression models adjusted for age, sex (MAP, CHAP only), study center and randomization status (WHIMS only). See Supplementary Table S4 for additional covariate details. T1, T2, T3 indicate tertile 1, tertile 2 and tertile 3 MIND adherence scores, respectively. ^2^ Basic + education, income, global cognition score, late-life cognitive activity (MAP, CHAP only), history of diabetes, hypertension, stroke and heart disease; smoking, calorie intake, BMI, depressive symptoms and physical activity.

**Table 3:** MIND score and cognitive decline.

Model	MAP		CHAP-White		CHAP-Black	
	β (95% CI)	*p*	β (95% CI)	*p*	β (95% CI)	*p*
Basic ^1^						
MIND T1	Ref.		Ref.		Ref.	
MIND T2	0.009 (−0.01, 0.03)	0.39	0.001 (−0.01, 0.01)	0.78	0.001 (−0.01, 0.01)	0.78
MIND T3	0.04 (0.02, 0.06)	0.0004	0.001(−0.01, 0.01)	0.77	−0.001 (−0.01, 0.01)	0.85
Trend	0.008 (0.003, 0.01)	0.002	0.0002 (−0.002, 0.003)	0.89	0.001 (−0.002, 0.003)	0.54
Full ^2^						
MIND T1	Ref.		Ref.		Ref.	
MIND T2	0.006 (−0.01, 0.02)	0.50	0.0001 (−0.01, 0.01)	0.99	0.0003 (−0.01, 0.01)	0.95
MIND T3	0.03 (0.01, 0.05)	0.001	−0.0008(−0.01, 0.01)	0.89	−0.003 (−0.01, 0.01)	0.51
Trend	0.006 (0.003, 0.01)	0.002	−0.0004 (−0.003, 0.002)	0.78	−0.00002 (−0.003, 0.003)	0.99

^1^ Results from mixed linear models adjusted for age and sex. See Table S4 for additional covariate details. T1, T2, T3 indicate tertile 1, tertile 2 and tertile 3 MIND adherence scores, respectively. Coefficients reflect change in cognitive function; a negative (positive) value corresponds to a decline (improvement) in cognitive function. ^2^ Basic + education, income, late-life cognitive activity, history of diabetes, hypertension, stroke and heart disease; smoking, calorie intake, BMI, depressive symptoms and physical activity.

**Table 4:** GS and risk of all-cause dementia ^1^.

GS	MAP		WHIMS		CHAP-White		CHAP-Black	
	HR (95% CI)	*p*	HR (95% CI)	*p*	OR (95% CI)	*p*	OR (95% CI)	*p*
GS_AD_								
T1	Ref.		Ref.		Ref.		Ref.	
T2	1.12 (0.80, 1.58)	0.51	1.28 (1.16, 1.41)	<0.001	1.55 (0.66, 3.66)	0.32	2.41 (0.82, 7.12)	0.11
T3	1.81 (1.31, 2.51)	0.0003	1.43 (1.30, 1.58)	<0.001	1.55 (0.55, 4.34)	0.41	0.77 (0.28, 2.11)	0.61
Trend	1.10 (1.05, 1.15)	<0.0001	1.05 (1.04, 1.06)	<0.0001	1.09 (0.92, 1.30)	0.32	1.06 (0.94, 1.20)	0.32
GS_AD-I_								
T1	Ref.		Ref.		Ref.		Ref.	
T2	1.45 (1.03, 2.02)	0.03	0.99 (0.89, 1.09)	0.764	1.12 (0.50, 2.53)	0.78	1.84 (0.76, 4.46)	0.18
T3	1.43 (1.03, 1.99)	0.03	1.11 (1.01, 1.23)	0.026	1.86 (0.74, 4.71)	0.19	0.66 (0.25, 1.77)	0.41
Trend	1.06 (1.00, 1.14)	0.06	1.02 (1.01, 1.04)	0.014	1.15 (0.96, 1.39)	0.14	0.88 (0.73, 1.07)	0.20
GS_AD-C_								
T1	Ref.		Ref.		Ref.		Ref.	
T2	0.86 (0.62, 1.20)	0.38	0.99 (0.90, 1.10)	0.911	1.01 (0.41, 2.45)	0.99	1.29 (0.41, 4.04)	0.66
T3	1.08 (0.79, 1.47)	0.64	1.25 (1.13, 1.37)	<0.0001	2.08 (0.82, 5.27)	0.12	0.57 (0.21, 1.55)	0.27
Trend	1.06 (0.93, 1.22)	0.36	1.08 (1.03, 1.12)	0.001	1.54 (0.86, 2.74)	0.15	1.06 (0.81, 1.38)	0.68

^1^ Results from Cox proportional hazard (HR) or logistic (OR) regression models adjusted for age, sex (MAP, CHAP only), study center and randomization status (WHIMS only) and genotype platform (MAP) or PCs (CHAP, WHIMS). T1, T2, T3 indicate tertile 1, tertile 2 and tertile 3 GS scores, respectively.

**Table 5:** Gene × MIND Interaction Tests ^1^.

Outcome	Interaction Term	MAP		WHIMS		CHAP (White)		CHAP (Black)		Meta-Analysis	
		β (SE)	*p*	β (SE)	*p*	β (SE)	*p*	β (SE)	*p*	β (SE)	*p*
All-cause dementia	GS_AD_ × MIND	−0.001 (0.01)	0.009	0.008 (0.004)	0.08	−0.02 (0.04)	0.63	0.02 (0.04)	0.52	0.007 (0.004)	0.07
	GS_AD-I_ × MIND	−0.009 (0.02)	0.67	−0.005 (0.006)	0.47	−0.02 (0.05)	0.61	0.08 (0.05)	0.13	−0.004 (0.006)	0.44
	GS_AD-C_ × MIND	0.006 (0.04)	0.88	−0.03 (0.01)	0.07	−0.07 (0.11)	0.51	0.04 (0.10)	0.70	−0.02 (0.01)	0.02
	*APOE* × MIND	0.12 (0.09)	0.17	−0.13 (0.03)	<0.0001	0.42 (0.22)	0.05	−0.08 (0.33)	0.81	−0.10 (0.03)	0.0006
Alzheimer’s disease	GS_AD_ × MIND	−0.001 (0.01)	0.004	n/a	n/a	−0.02 (0.04)	0.65	0.02 (0.04)	0.55	−0.0009 (0.009)	0.92
	GS_AD-I_ × MIND	−0.006 (0.02)	0.76	n/a	n/a	−0.02 (0.05)	0.69	0.08 (0.06)	0.12	−0.0002 (0.02)	0.99
	GS_AD-C_ × MIND	0.01 (0.04)	0.07	n/a	n/a	−0.05 (0.12)	0.69	0.04 (0.11)	0.72	0.008 (0.04)	0.83
	*APOE* × MIND	0.14 (0.09)	0.12	n/a	n/a	0.44 (0.23)	0.05	−0.09 (0.34)	0.80	0.16 (0.08)	0.04
Cognitive decline	GS_AD_ × MIND × time	0.001 (0.0008)	0.24	n/a	n/a	0.001 (0.0004)	0.23	−0.0006 (0.0005)	0.19	0.0005 (0.0003)	0.12
	GS_AD-I_ × MIND × time	0.0008 (0.001)	0.49	n/a	n/a	−0.0001 (0.001)	0.93	−0.002 (0.0008)	0.008	−0.0007 (0.0005)	0.20
	GS_AD-C_ × MIND × time	0.003 (0.002)	0.28	n/a	n/a	0.001 (0.001)	0.57	−0.003 (0.001)	0.016	−0.0006 (0.0007)	0.40
	*APOE* × MIND × time	−0.002 (0.006)	0.69	n/a	n/a	0.0001 (0.003)	0.97	0.006 (0.003)	0.04	0.003 (0.002)	0.21

^1^ Results from basic models (adjusted for age and sex).

## Data Availability

MAP data can be requested at https://www.radc.rush.edu. WHIMS data can be requested upon approval at whi.org. CHAP data are available to external investigators upon approval by CHAP research committee and Rush University http://www.riha.rush.edu.

## Supplementary Materials

The following are available online at www.mdpi.com/xxx/s1www.mdpi.com: Figure S1: MIND adherence score distributions by cohort, Supplementary Table S1: The Mediterranean-DASH Diet Intervention for Neurodegenerative Delay (MIND) Adherence Score Derivation, Supplementary Table S2: Cognitive Tests Used in Cohorts, Supplementary Table S3: Statistical Model Covariates for Dementia and Cognitive Decline Analysis, Supplementary Table S4: Analytical Samples, Supplementary Table S5: Late-Onset Alzheimer’s Disease Risk Loci, Supplementary Table S6: GS and Risk of Alzheimer’s Dementia, Supplementary Table S7: GS and Cognitive Decline [55,56,57,58,59,60,61,62,63].

## References

[1] Alzheimer’s Association Trajectory Report. https://www.alz.org/help-support/resources/publications/trajectory_report.

[2] Aridi Y., Walker J., Wright O. (2017). The association between the Mediterranean dietary pattern and cognitive health: A systematic review. Nutrients.

[3] Morris M.C. (2012). Nutritional determinants of cognitive aging and dementia. Proc. Nutr. Soc..

[4] Jansen I., Savage J., Watanabe K., Bryois J., Williams D., Steinberg S., Sealock J., Karlsson I., Hägg S., Athanasiu L. (2019). Genome-wide meta-analysis identifies new loci and functional pathways influencing Alzheimer’s disease risk. Nat. Genet..

[5] Desikan R.S., Schork A.J., Wang Y., Thompson W.K., Dehghan A., Ridker P.M., Chasman D.I., McEvoy L.K., Holland D., Chen C.-H. (2015). Polygenic overlap between c-reactive protein, plasma lipids, and Alzheimer disease. Circulation.

[6] Chen J., Xie C., Zhao Y., Li Z., Xu P., Yao L. (2016). Gene expression analysis reveals the dysregulation of immune and metabolic pathways in Alzheimer’s disease. Oncotarget.

[7] Sureda A., Bibiloni M.D.M., Julibert A., Bouzas C., Argelich E., Llompart I., Pons A., Tur J.A. (2018). Adherence to the Mediterranean diet and inflammatory markers. Nutrients.

[8] Grosso G., Mistretta A., Frigiola A., Gruttadauria S., Biondi A., Basile F., Vitaglione P., D’Orazio N., Galvano F. (2014). Mediterranean diet and cardiovascular risk factors: A systematic review. Crit. Rev. Food Sci. Nutr..

[9] Toledo E., Wang D.D., Ruiz-Canela M., Clish C.B., Razquin C., Zheng Y., Guasch-Ferre M., Hruby A., Corella D., Gomez-Gracia E. (2017). Plasma lipidomic profiles and cardiovascular events in a randomized intervention trial with the Mediterranean diet. Am. J. Clin. Nutr..

[10] Nishida Y., Ito S., Ohtsuki S., Yamamoto N., Takahashi T., Iwata N., Jishage K.-I., Yamada H., Sasaguri H., Yokota S. (2009). Depletion of vitamin E increases amyloid beta accumulation by decreasing its clearances from brain and blood in a mouse model of Alzheimer disease. J. Biol. Chem..

[11] Calon F., Lim G.P., Yang F., Morihara T., Teter B., Ubeda O., Rostaing P., Triller A., Salem N., Ashe K.H. (2004). Docosahexaenoic acid protects from dendritic pathology in an Alzheimer’s disease mouse model. Neuron.

[12] Hussain T., Tan B., Yin Y., Blachier F., Tossou M.C.B., Rahu N. (2016). Oxidative stress and inflammation: What polyphenols can do for us?. Oxidative Med. Cell. Longev..

[13] Lim S.Y., Suzuki H. (2000). Effect of dietary docosahexaenoic acid and phosphatidylcholine on maze behavior and fatty acid composition of plasma and brain lipids in mice. Int. J. Vitam. Nutr. Res..

[14] Evans D.A., Bennett D.A., Wilson R.S., Bienias J.L., Morris M.C., Scherr P.A., Hebert L.E., Aggarwal N., Beckett L.A., Joglekar R. (2003). Incidence of Alzheimer disease in a biracial urban community: Relation to apolipoprotein e allele status. Arch. Neurol..

[15] Rajan K.B., Weuve J., Barnes L.L., Wilson R.S., Evans D.A. (2019). Prevalence and incidence of clinically diagnosed Alzheimer’s disease dementia from 1994 to 2012 in a population study. Alzheimer’s Dement..

[16] Bennett D.A., Schneider J.A., Buchman A.S., Barnes L.L., Boyle P.A., Wilson R.S. (2012). Overview and findings from the rush memory and aging project. Curr. Alzheimer Res..

[17] Shumaker S.A., Legault C., Rapp S.R., Thal L., Wallace R.B., Ockene J.K., Hendrix S.L., Jones B.N., Assaf A.R., Jackson R.D. (2003). Estrogen plus progestin and the incidence of dementia and mild cognitive impairment in postmenopausal women: The women’s health initiative memory study: A randomized controlled trial. JAMA.

[18] Anderson G.L., Limacher M. (2004). Effects of conjugated equine estrogen in postmenopausal women with hysterectomy: The women’s health initiative randomized controlled trial. J. Am. Med. Assoc..

[19] Rapp S.R., Espeland M.A., Shumaker S.A., Henderson V.W., Brunner R.L., Manson J.E., Gass M.L., Stefanick M.L., Lane D.S., Hays J. (2003). Effect of estrogen plus progestin on global cognitive function in postmenopausal women: The women’s health initiative memory study: A randomized controlled trial. JAMA.

[20] Rossouw J.E., Anderson G.L., Prentice R.L., LaCroix A.Z., Kooperberg C., Stefanick M.L., Jackson R.D., Beresford S.A.A., Howard B.V., Johnson K.C. (2002). Risks and benefits of estrogen plus progestin in healthy postmenopausal women: Principal results from the women’s health initiative randomized controlled trial. J. Am. Med. Assoc..

[21] Espeland M.A., Rapp S.R., Shumaker S.A., Brunner R., Manson J.E., Sherwin B.B., Hsia J., Margolis K.L., Hogan P.E., Wallace R. (2004). Conjugated equine estrogens and global cognitive function in postmenopausal women: Women’s health initiative memory study. JAMA.

[22] Shumaker S.A., Legault C., Kuller L., Brunner R., Manson J.E., Sherwin B.B., Hsia J., Margolis K.L., Hogan P.E., Wallace R. (2004). Conjugated equine estrogens and incidence of probable dementia and mild cognitive impairment in postmenopausal women: Women’s health initiative memory study. JAMA.

[23] Morris M.C., Tangney C.C., Wang Y., Sacks F.M., Bennett D.A., Aggarwal N.T. (2015). Mind diet associated with reduced incidence of Alzheimer’s disease. Alzheimer’s Dement..

[24] Teng E.L., Chui H.C. (1987). The modified mini-mental state (3ms) examination. J. Clin. Psychiatry.

[25] Rapp S.R., Legault C., Espeland M.A., Resnick S.M., Hogan P.E., Coker L.H., Dailey M., Shumaker S.A. (2012). Validation of a cognitive assessment battery administered over the telephone. J. Am. Geriatr. Soc..

[26] Ellis R.J., Jan K., Kawas C., Koller W.C., Lyons K.E., Jeste D.V., Hansen L.A., Thal L.J. (1998). Diagnostic validity of the dementia questionnaire for Alzheimer disease. Arch. Neurol..

[27] Bienias J.L., Beckett L.A., Bennett D.A., Wilson R.S., Evans D.A. (2003). Design of the Chicago health and aging project (CHAP). J. Alzheimers Dis..

[28] Bienias J.L., Kott P.S., Evans D.A. (2003). Applying the delete-a-group jackknife variance estimator to analyses of data from a complex longitudinal survey. Proceedings of the Annual Meeting of the American Statistical Association, Section on Survey Research Methods.

[29] McKhann G., Drachman D., Folstein M., Katzman R., Price D., Stadlan E.M. (1984). Clinical diagnosis of Alzheimer’s disease: Report of the NINCDS-ADRDA Work Group under the auspices of Department of Health and Human Services Task Force on Alzheimer’s Disease. Neurology.

[30] Bennett D.A., Schneider J.A., Aggarwal N.T., Arvanitakis Z., Shah R.C., Kelly J.F., Fox J.H., Cochran E.J., Arends D., Treinkman A.D. (2006). Decision rules guiding the clinical diagnosis of Alzheimer’s disease in two community-based cohort studies compared to standard practice in a clinic-based cohort study. Neuroepidemiology.

[31] De Jager P.L., Shulman J.M., Chibnik L.B., Keenan B.T., Raj T., Wilson R.S., Yu L., Leurgans S.E., Tran D., Aubin C. (2012). A genome-wide scan for common variants affecting the rate of age-related cognitive decline. Neurobiol. Aging.

[32] Dumitrescu L., Mahoney E.R., Mukherjee S., Lee M.L., Bush W.S., Engelman C.D., Lu Q., Fardo D.W., Trittschuh E.H., Mez J. (2020). Genetic variants and functional pathways associated with resilience to Alzheimer’s disease. Brain.

[33] Hayden K.M., Wang Y., Beavers D., Chen J.-C., Espeland M.A., Ford C.N., Harrington L.B., He K., Jensen M.K., Johnson K.C. (2017). The mind diet and incident dementia: Findings from the women’s health initiative memory study. Alzheimer’s Dement. J. Alzheimer’s Assoc..

[34] Chouraki V., Reitz C., Maury F., Bis J.C., Bellenguez C., Yu L., Jakobsdottir J., Mukherjee S., Adams H.H., Choi S.H. (2016). Evaluation of a genetic risk score to improve risk prediction for Alzheimer’s disease. J. Alzheimer’s Dis..

[35] Driscoll I., Snively B.M., Espeland M.A., Shumaker S.A., Rapp S.R., Goveas J.S., Casanova R.L., Wactawski-Wende J., Manson J.E., Rossom R. (2019). A candidate gene study of risk for dementia in older, postmenopausal women: Results from the women’s health initiative memory study. Int. J. Geriatr. Psychiatry.

[36] Rajan K.B., Barnes L.L., Wilson R.S., Weuve J., McAninch E.A., Evans D.A. (2019). Apolipoprotein E genotypes, age, race, and cognitive decline in a population sample. J. Am. Geriatr. Soc..

[37] Schoenfeld D. (1982). Partial residuals for the proportional hazards regression model. Biometrika.

[38] Rajan K.B., Barnes L.L., Wilson R.S., Weuve J., McAninch E.A., Evans D.A. (2018). Blood pressure and risk of incident Alzheimer’s disease dementia by antihypertensive medications and APOE ε4 allele. Ann. Neurol..

[39] Samuelsson J., Najar J., Wallengren O., Kern S., Wetterberg H., Mellqvist Fässberg M., Zetterberg H., Blennow K., Lissner L., Rothenberg E. (2022). Interactions between dietary patterns and genetic factors in relation to incident dementia among 70-year-olds. Eur. J. Nutr..

[40] Hossain S., Beydoun M.A., Weiss J., Kuczmarski M.F., Evans M.K., Zonderman A.B. (2020). Longitudinal associations between dietary quality and Alzheimer’s disease genetic risk on cognitive performance among African American adults. Br. J. Nutr..

[41] Cornelis M.C., Hu F.B. (2012). Gene-environment interactions in the development of type 2 diabetes: Recent progress and continuing challenges. Annu. Rev. Nutr..

[42] Tangney C.C., Kwasny M.J., Li H., Wilson R.S., Evans D.A., Morris M.C. (2011). Adherence to a Mediterranean-type dietary pattern and cognitive decline in a community population. Am. J. Clin. Nutr..

[43] Duplantier S.C., Gardner C.D. (2021). A critical review of the study of neuroprotective diets to reduce cognitive decline. Nutrients.

[44] De Crom T.O.E., Mooldijk S.S., Ikram M.K., Ikram M.A., Voortman T. (2022). Mind diet and the risk of dementia: A population-based study. Alzheimer’s Res. Ther..

[45] Rovio S., Kåreholt I., Helkala E.-L., Viitanen M., Winblad B., Tuomilehto J., Soininen H., Nissinen A., Kivipelto M. (2005). Leisure-time physical activity at midlife and the risk of dementia and Alzheimer’s disease. Lancet Neurol..

[46] Anttila T., Helkala E.-L., Viitanen M., Kåreholt I., Fratiglioni L., Winblad B., Soininen H., Tuomilehto J., Nissinen A., Kivipelto M. (2004). Alcohol drinking in middle age and subsequent risk of mild cognitive impairment and dementia in old age: A prospective population based study. Bmj.

[47] Laitinen M.H., Ngandu T., Rovio S., Helkala E.L., Uusitalo U., Viitanen M., Nissinen A., Tuomilehto J., Soininen H., Kivipelto M. (2006). Fat intake at midlife and risk of dementia and Alzheimer’s disease: A population-based study. Dement. Geriatr. Cogn. Disord..

[48] Kivipelto M., Rovio S., Ngandu T., Kåreholt I., Eskelinen M., Winblad B., Hachinski V., Cedazo-Minguez A., Soininen H., Tuomilehto J. (2008). Apolipoprotein E ɛ4 magnifies lifestyle risks for dementia: A population-based study. J. Cell. Mol. Med..

[49] Barberger-Gateau P., Raffaitin C., Letenneur L., Berr C., Tzourio C., Dartigues J.-F., Alpérovitch A. (2007). Dietary patterns and risk of dementia the three-city cohort study. Neurology.

[50] Podewils L.J., Guallar E., Kuller L.H., Fried L.P., Lopez O.L., Carlson M., Lyketsos C.G. (2005). Physical activity, APOE genotype, and dementia risk: Findings from the cardiovascular health cognition study. Am. J. Epidemiol..

[51] Huang T.L., Zandi P., Tucker K., Fitzpatrick A., Kuller L., Fried L., Burke G., Carlson M. (2005). Benefits of fatty fish on dementia risk are stronger for those without APOE ε4. Neurology.

[52] Luchsinger J.A., Tang M.X., Siddiqui M., Shea S., Mayeux R. (2004). Alcohol intake and risk of dementia. J. Am. Geriatr. Soc..

[53] Licher S., Ahmad S., Karamujić-Čomić H., Voortman T., Leening M.J.G., Ikram M.A., Ikram M.K. (2019). Genetic predisposition, modifiable-risk-factor profile and long-term dementia risk in the general population. Nat. Med..

[54] Lourida I., Hannon E., Littlejohns T.J., Langa K.M., Hyppönen E., Kuźma E., Llewellyn D.J. (2019). Association of lifestyle and genetic risk with incidence of dementia. JAMA.

[55] Hollingworth P., Harold D., Sims R., Gerrish A., Lambert J.-C., Carrasquillo M.M., Abraham R., Hamshere M.L., Pahwa J.S., Moskvina V. (2011). Common variants at ABCA7, MS4A6A/MS4A4E, EPHA1, CD33 and CD2AP are associated with Alzheimer’s disease. Nat. Genet..

[56] Lambert J.C., Ibrahim-Verbaas C.A., Harold D., Naj A.C., Sims R., Bellenguez C., DeStafano A.L., Bis J.C., Beecham G.W., Grenier-Boley B. (2013). Meta-analysis of 74,046 individuals identifies 11 new susceptibility loci for Alzheimer’s disease. Nat. Genet..

[57] Naj A.C., Jun G., Beecham G.W., Wang L.S., Vardarajan B.N., Buros J., Gallins P.J., Buxbaum J.D., Jarvik G.P., Crane P.K. (2011). Common variants at MS4A4/MS4A6E, CD2AP, CD33 and EPHA1 are associated with late-onset Alzheimer’s disease. Nat. Genet..

[58] Herold C., Hooli B.V., Mullin K., Liu T., Roehr J.T., Mattheisen M., Parrado A.R., Bertram L., Lange C., Tanzi R.E. (2016). Family-based association analyses of imputed genotypes reveal genome-wide significant association of Alzheimer’s disease with OSBPL6, PTPRG, and PDCL3. Mol. Psychiatry.

[59] Lambert J.C., Heath S., Even G., Campion D., Sleegers K., Hiltunen M., Combarros O., Zelenika D., Bullido M.J., Tavernier B. (2009). Genome-wide association study identifies variants at CLU and CR1 associated with Alzheimer’s disease. Nat. Genet..

[60] Jun G., Ibrahim-Verbaas C.A., Vronskaya M., Lambert J.C., Chung J., Naj A.C., Kunkle B.W., Wang L.S., Bis J.C., Bel-lenguez C. (2016). A novel Alzheimer disease locus located near the gene encoding tau protein. Mol. Psychiatry.

[61] Jun G.R., Chung J., Mez J., Barber R., Beecham G.W., Bennett D.A., Buxbaum J.D., Byrd G.S., Carrasquillo M.M., Crane P.K. (2017). Transethnic genome-wide scan identifies novel Alzheimer’s disease loci. Alzheimer’s Dement..

[62] Jiang Q., Jin S., Jiang Y., Liao M., Feng R., Zhang L., Liu G., Hao J. (2016). Alzheimer’s Disease Variants with the Genome-Wide Significance are Significantly Enriched in Immune Pathways and Active in Immune Cells. Mol. Neurobiol..

[63] Kunkle B.W., Grenier-Boley B., Sims R., Bis J.C., Damotte V., Naj A.C., Boland A., Vronskaya M., Van Der Lee S.J., Amlie-Wolf A. (2019). Genetic meta-analysis of diagnosed Alzheimer’s disease identifies new risk loci and implicates Aβ, tau, immunity and lipid processing. Nat. Genet..

