# Supplementary material for: Adherence to MIND Diet, Genetic Susceptibility, and Incident Dementia in Three US Cohorts
Source: Nutrients. 2022 Jul 3;14(13):2759. doi: 10.3390/nu14132759 (PMC9268772; doi:10.3390/nu14132759)
Supplement: Supplementary file 1 [file nutrients-14-02759-s001.zip › nutrients-1778555-supplementary.pdf]

Figure S1. MIND adherence score distributions by cohort

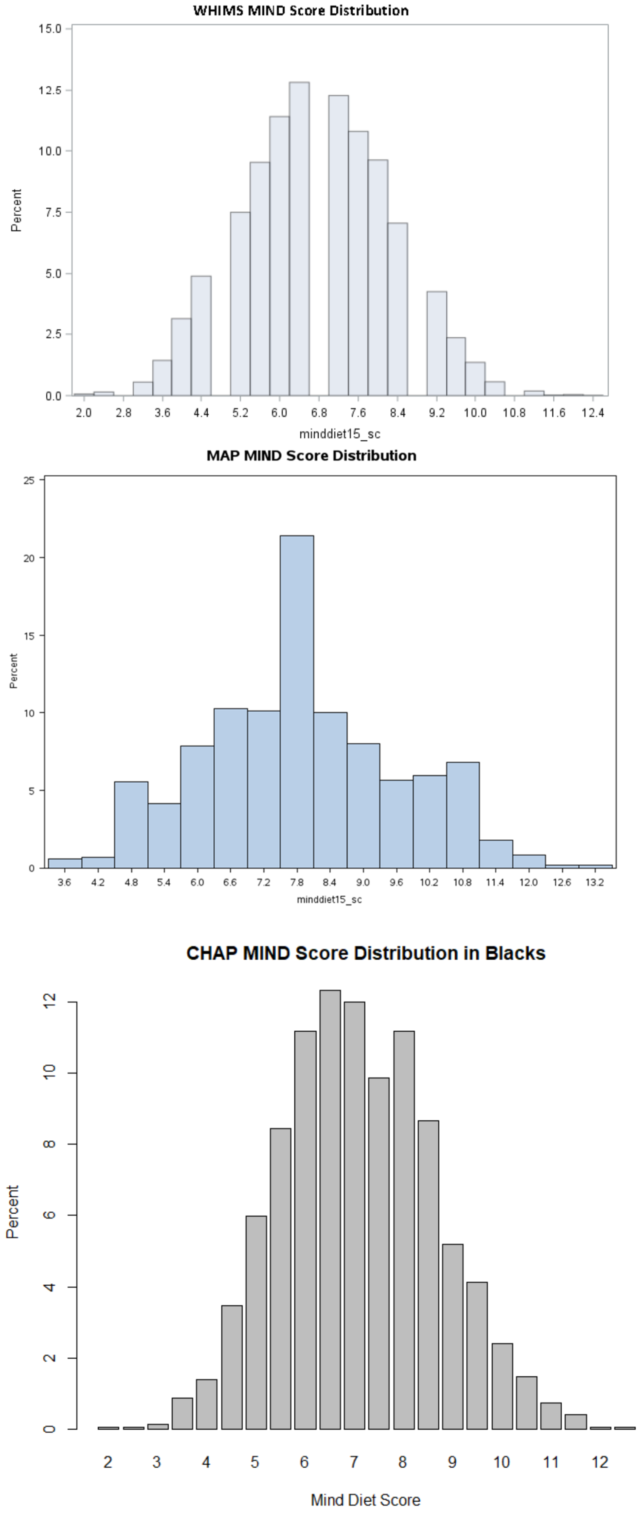

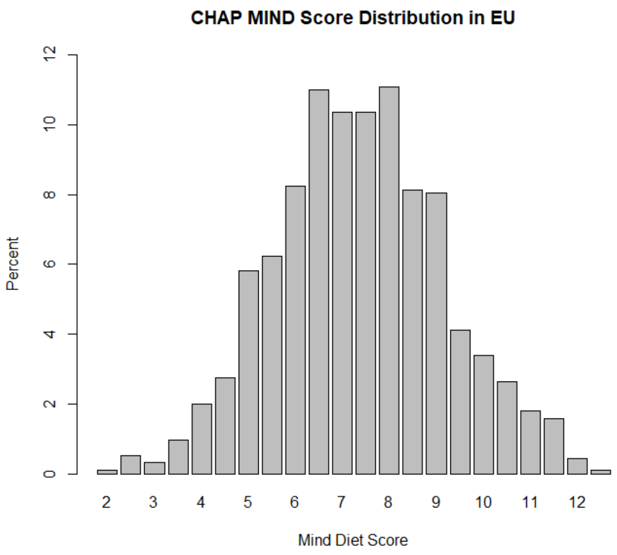
CHAP MIND Score Distribution in EU
Percent
0
2
4
6
8
10
12
2
3
4
5
6
7
8
9
10
11
12
Mind Diet Score

Table S1. The Mediterranean-DASH Diet Intervention for Neurodegenerative Delay (MIND) Adherence Score Derivation

MIND component	# servings (serving size)* for max point =1		
	MAP	WHIMS	CHAP
Green leafy vegetables	≥6 /wk (1/2 - 1 cup)	≥6 /wk (1/2 - 1 cup)	≥6 /wk (1/2 - 1 cup)
Other vegetables	≥1 /d (1/2 cup)	≥1 /d (1/2 cup)	≥1 /d (1/2 cup)
Nuts (mixed nuts, peanut butter)	≥5 /wk (1 oz)	≥5 /wk (1 oz)	≥5 /wk (1 oz)
Berries	≥2 /wk (1/2 cup)	≥2 /wk (1/2 cup)	≥2 /wk (1/2 cup)
Beans/legumes	>3 /wk (1/2 cup each)	>3 /wk (1/2 cup each)	>3 /wk (1/2 cup each)
Whole grains	≥3 /d†	≥3 /d†	≥3 /d†
Fish (not fried)	≥1 /wk (3-5 oz)	≥1 /wk (3-5 oz)	≥1 /wk (3-5 oz)
Poultry (not fried, white meat, skinless)	≥2 /wk (3-5 oz each)	≥2 /wk (3-5 oz each)	≥2 /wk (3-5 oz each)
Extra virgin olive oil	Primary cooking oil: Olive/canola oil	Primary cooking oil Olive/canola oil	Primary cooking oil Olive/canola oil
Red and processed meats	<4 /wk (3-5 oz each)	<4 /wk (3-5 oz each)	<4 /wk (3-5 oz each)
Butter and stick margarine	<1 tsp /d	≤1 /d (1 pat/tsp)	≤1 pats/d
Cheese	<1 /wk (1 oz)	<1 /wk (1 oz)	<1 /wk (1 oz)
Pastries, candy bars, sweets	<5 /wk	<5 /wk	<5 /wk
Fried foods and fast food	<1 /wk (1 meal)	<1 /wk (1 meal)	≤1 /wk (1 meal)
Wine	2-7/wk -1/d (~5 oz)	1 /d (~5 oz)	2-4/wk – 1/d

†MAP: 1 slice dark bread, ½ dark bagel, ½ c brown rice/pastas, wild rice, quinoa, barley, buckwheat, bulgur, farro, kamut, millet, oats, rye, spelt, ¾ c whole grain cereal. WHIMS: cold cereal including: granola; high fiber cold cereal; whole grain cold cereal; and dark bread (bagel/roll/pita/eng muff) CHAP: hot cereal like oatmeal or grits, dark bread, other grains like kasha, couscous or bulgar

*Only food item portions resulting in 1 point are shown; 0 or 0.5 points are also given.

Table S2. Cognitive Tests Used in Cohorts

Test	MAP	CHAP	WHIMS
**Domain: Episodic Memory**			
Word list memory	x‡		x*
Word list recall	x‡		x*
Word list recognition	x‡		x*
East Boston story immediate	x‡	x‡	
East Boston story delayed	x‡	x‡	
Logical Memory Ia immediate	x‡		
Logical Memory IIa delayed	x‡		
**Domain: Semantic Memory**			
Boston Naming Test	x‡		x*
Verbal fluency	x‡		
Reading test	x‡		
**Domain: Working Memory**			
Digit span forward	x‡		
Digit span backward	x‡		
Digit ordering	x‡		
**Domain: Perceptual Speed**			
Symbol Digit Modalities Test	x‡	x‡	
Number comparison	x‡		
Stroop word reading	x‡		
Stroop color naming	x‡		
**Domain: Visuospatial Ability**			
Judgment of line orientation	x‡		
Standard progressive matrices	x‡		
**Domain: Praxis**			
Constructional praxis			x*
**Domain: Executive Function**			
Trail Making Test A and B			x*
**Global Cognitive Function**			
Mini-Mental State Examination	x	x‡	
Modified Mini-Mental State Examination			x
Modified Telephone Interview for Cognitive Status			x†

*WHIMS (1996-2008): test completed only if participant met specific 3MSE score threshold
†WHIMS-ECHO, 2008-2021.
‡These tests were used in composite scores of global cognition (see Methods)

Table S3. Statistical Model Covariates for Dementia and Cognitive Decline Analysis

Model	Set	MAP (white)	WHIMS (white women)	CHAP (white)	CHAP (black)
**Basic**	**Basic**	MIND score(T)<br>Age(C)<br>Sex(D)<br>Genotyping platform(D)[3]	MIND score(T)<br>Age(C)<br>Region (4 categories)<br>Randomization status (4 categories)<br>GWAS set (D)[3]<br>10 principle components(C)[3]	MIND score(T)<br>Age(C)<br>Sex(D)<br>10 principle components(C)[3]	MIND score(T)<br>Age(C)<br>Sex(D)<br>10 principle components(C)[3]
	Cognitive Reserve (CogRes)	**Basic**+<br>Years of education(C)<br>Late-life cognitive activity(Q) [4]<br>Global cognition score(C)[2]<br>Income(5 categories)[1]	**Basic**+<br>Education (3 categories)<br>Global cognition score (C) [2]<br>Income (6 categories)	**Basic**+<br>Years of education(C)<br>Late-life cognitive activity(C)<br>Global cognition score(C)[2]<br>Income(C)	**Basic**+<br>Years of education(C)<br>Late-life cognitive activity(C)<br>Global cognition score(C)[2]<br>Income(C)
	Disease	**Basic**+<br>History of hypertension(D)[4]<br>History of diabetes(D) [4]<br>History of heart disease(D) [4]<br>History of stroke(D) [4]	**Basic**+<br>History of hypertension(D)<br>History of diabetes(D)<br>History of heart disease(D)<br>History of stroke(D)	**Basic**+<br>History of hypertension(D)<br>History of diabetes(D)<br>History of heart disease(D)<br>History of stroke(D)	**Basic**+<br>History of hypertension(D)<br>History of diabetes(D)<br>History of heart disease(D)<br>History of stroke(D)
	Lifestyle	**Basic**+<br>Smoking(3 categories)<br>Calories(C)<br>CESD score(T) [4]<br>Physical activity(Q) [4]<br>BMI(5 categories) [1, 4]	**Basic**+<br>Smoking(3 categories)<br>Calories(C)<br>Depression score (T)<br>Physical activity(T)<br>BMI(3 categories)	**Basic**+<br>Smoking(3 categories)<br>Calories(C)<br>CESD score(C)<br>Physical activity(C)<br>BMI(5 categories) [1]	**Basic**+<br>Smoking(3 categories)<br>Calories(C)<br>CESD score(C)<br>Physical activity(C)<br>BMI(5 categories) [1]
Full	Full	All of the above	All of the above	All of the above	All of the above

(C) continuous, (D) dichotomous, (T) tertiles, (Q) quartiles,

[1]Includes a 'missing' category.

[2]Not included for analysis of cognitive decline.

[3]Used in genetic analysis only. For MAP, the genetic sample is of European-ancestry only. For WHIMS, genetic analyses are restricted to genetically-inferred European ancestry (89% of WHIMS). For CHAP, genetic analyses are performed separately for genetically inferred European and African American.

[4]Time-varying covariates for analysis of cognitive decline.

Table S4. Analytical Samples

	MAP	WHIMS	CHAP-White	CHAP-Black
**Initial n**<br>Non-missing baseline outcome data (dementia/cognition score), MIND score and model 1 covariates	1054	6851	2542	4314
Exclude participants with missing genetic data	-224	-1417	-1565	-2651
Exclude participants with <1 follow-up assessment (i.e. all participants require at least a baseline visit and 1 follow-up)	-63	-126	-31	-160
Exclude prevalent (baseline) cases of dementia	-42	0	0	0
**Final n**	725	5308	946	1503

Table S5. Late-Onset Alzheimer's Disease Risk Loci[1-10]

Closest Gene	Chr:Pos	SNP_EA	OA	EAF		Effect OR*	Effect β	GS_{AD}	GS_{AD-I}	GS_{AD-C}
				EUR	AFR					
CR1	1:207692049	rs6656401_A	G	0.20	0.01	1.18	0.16	x	x	
BIN1	2:127892810	rs6733839_T	C	0.41	0.42	1.22	0.19	x		
INPP5D	2:234068476	rs35349669_T	C	0.48	0.10	1.08	0.07	x	x	
CLNK	4:11026028	rs6448453_A	G	0.25	0.10	1.09	0.09	x	x	
MEF2C	5:88223420	rs190982_A	G	0.63	0.92	1.08	0.08	x	x	
HLA-DRB1/5	6:32578530	rs9271192_C (rs111418223_C)	A	0.27	0.23	1.11	0.10	x	x	
CD2AP	6:47487762	rs10948363_G	A	0.27	0.27	1.1	0.10	x		
NME8	7:37841534	rs2718058_A	G	0.64	0.49	1.08	0.07	x		
ZCWPW1	7:100004446	rs1476679_T	C	0.69	0.98	1.1	0.08	x		
EPHA1	7:143110762	rs11771145_G	A	0.63	0.43	1.11	0.10	x	x	
PTK2B	8:27195121	rs28834970_C	T	0.35	0.22	1.1	0.10	x		
CLU	8:27467686	rs9331896_T	C	0.60	0.38	1.16	0.15	x	x	x
ECHDC3	10:11720308	rs7920721_G	A	0.36	0.11	1.08	0.08	x		
CELF1	11:47557871	rs10838725_C	T	0.27	0.02	1.08	0.08	x		
MS4A6A	11:59923508	rs983392_A	G	0.58	0.97	1.16	0.11	x	x	
PICALM	11:85867875	rs10792832_G	A	0.62	0.91	1.34	0.13	x		
SORL1	11:121435587	rs11218343_T	C	0.96	0.10	1.3	0.27	x		x
FERMT2	14:53400629	rs17125944_C	T	0.08	0.06	1.14	0.12	x		
SLC24A4	14:92926952	rs10498633_G	T	0.79	0.88	1.01	0.10	x		x
ADAM10	15:59045774	rs593742_A	G	0.64	0.21	1.04	0.08	x		
IQCK	16:19808163	rs7185636_T	C	0.85	0.09	1.09	0.09	x		
ABCA7	19:1063443	rs4147929_A	G	0.17	0.02	1.22	0.13	x		x
CD33	19:51727962	rs3865444_C	A	0.69	0.93	1.10	0.10	x	x	
CASS4	20:55018260	rs7274581_T	C	0.92	0.73	1.14	0.14	x		
ADAMTS1	21:28156856	rs2830500_C	A	0.70	0.91	1.07	0.07	x	x	

AFR: African ancestry, EA: effect allele, EAF: EA frequency, EUR: European ancestry, GS_{AD}: AD predisposition genetic score (I: immune response; C: cholesterol metabolism), OA: other allele; OR: odds ratio. *As reported by the two largest GWAS to date[1, 3].

Table S6. GS and Risk of Alzheimer's Dementia[1]

Genetic exposure	MAP		CHAP (White)		CHAP (Black)	
	HR (95% CI)	P	OR (95% CI)	P	OR (95% CI)	P
GS_{AD}						
T1	Ref.		Ref.		Ref.	
T2	1.04 (0.73, 1.47)	0.85	1.48 (0.60, 3.66)	0.39	2.38 (0.79, 7.18)	0.12
T3	1.72 (1.24, 2.39)	0.001	1.51 (0.52, 4.40)	0.45	0.72 (0.26, 2.04)	0.54
Trend	1.10 (1.05, 1.15)	0.0001	1.10 (0.92, 1.32)	0.31	1.06 (0.94, 1.21)	0.34
GS_{AD-I}						
T1	Ref.		Ref.		Ref.	
T2	1.34 (0.95, 1.89)	0.09	1.15 (0.49, 2.72)	0.74	1.84 (0.75, 4.50)	0.18
T3	1.37 (0.98, 1.91)	0.06	2.01 (0.77, 5.26)	0.15	0.63 (0.23, 1.71)	0.36
Trend	1.06 (0.99, 1.13)	0.10	1.18 (0.97, 1.43)	0.10	0.87 (0.72, 1.05)	0.15
GS_{AD-C}						
T1	Ref.		Ref.		Ref.	
T2	0.92 (066, 1.30)	0.65	0.99 (0.38, 2.54)	0.97	1.26 (0.39, 4.06)	0.70
T3	1.13 (0.82, 1.55)	0.45	2.27 (0.86, 5.97)	0.10	0.55 (0.20, 1.54)	0.25
Trend	1.08 (0.94, 1.23)	0.29	1.64 (0.88, 3.03)	0.12	1.06 (0.81, 1.39)	0.69
APOE						
Non-carrier	Ref.		Ref.		Ref.	
Carrier	2.01 (1.49, 2.71)	<.0001	1.90 (0.76, 4.72)	0.17	1.13 (0.42, 3.05)	0.81

[1]Results from Cox proportional hazard regression models adjusted for age, sex and genotype platform (MAP) or PCs (CHAP). T1, T2, T3 indicate tertile 1, tertile 2 and tertile 3 GS scores, respectively.

Table S7. GS and Cognitive Decline[1]

GS	MAP		CHAP-White		CHAP-Black	
	β (95% CI)	P	β (95% CI)	P	β (95% CI)	P
GS_{AD}						
T1	Ref.		Ref.		Ref.	
T2	-0.01 (-0.03, 0.006)	0.16	0.003 (-0.01,0.01)	0.64	-0.004 (-0.01,0.01)	0.44
T3	-0.04 (-0.06, -0.02)	<.0001	0.002 (-0.01,0.01)	0.67	-0.002 (-0.01,0.01)	0.65
Trend	-0.006 (-0.009, -0.004)	<.0001	-0.0002 (-0.002,0.001)	0.81	-0.0003 (-0.002,0.001)	0.72
GS_{AD-I}						
T1	Ref.		Ref.		Ref.	
T2	-0.03 (-0.05, -0.007)	0.007	-0.0001 (-0.01,0.01)	0.98	-0.003 (-0.01,0.01)	0.58
T3	-0.03 (-0.05, -0.01)	0.003	-0.0009 (-0.01,0.01)	0.88	0.002 (-0.007,0.012)	0.64
Trend	-0.005 (-0.009, -0.001)	0.01	-0.0006 (-0.003,0.001)	0.55	0.0005 (-0.002,0,003)	0.68
GS_{AD-C}						
T1	Ref.		Ref.		Ref.	
T2	0.008 (-0.01, 0.03)	0.46	-0.006 (-0.02,0.005)	0.27	-0.002 (-0.01,0.01)	0.67
T3	-0.007 (-0.03, 0.01)	0.52	-0.005 (-0.02,0.006)	0.39	0.001 (-0.01,0.01)	0.88
Trend	-0.005 (-0.01, 0.004)	0.28	-0.003 (-0.01,0.002)	0.27	0.001 (-0.003,0.005)	0.53

[1] Results from mixed linear models adjusted for age, sex and genotyping platform (MAP) or PCs (CHAP). Coefficients reflect change in cognitive function; a negative (positive) value corresponds to a decline (improvement) in cognitive function. T1, T2, T3 indicate tertile 1, tertile 2 and tertile 3 GS scores, respectively.

References

1. Jansen I, Savage J, Watanabe K, et al. Genome-wide meta-analysis identifies new loci and functional pathways influencing Alzheimer's disease risk. Nature genetics 2019.
2. Hollingworth P, Harold D, Sims R, et al. Common variants at ABCA7, MS4A6A/MS4A4E, EPHA1, CD33 and CD2AP are associated with Alzheimer's disease. Nat Genet 2011;43:429-435.
3. Lambert JC, Ibrahim-Verbaas CA, Harold D, et al. Meta-analysis of 74,046 individuals identifies 11 new susceptibility loci for Alzheimer's disease. Nat Genet 2013;45:1452-1458.
4. Naj AC, Jun G, Beecham GW, et al. Common variants at MS4A4/MS4A6E, CD2AP, CD33 and EPHA1 are associated with late-onset Alzheimer's disease. Nat Genet 2011;43:436-441.
5. Herold C, Hooli BV, Mullin K, et al. Family-based association analyses of imputed genotypes reveal genome-wide significant association of Alzheimer's disease with OSBPL6, PTPRG, and PDCL3. Molecular psychiatry 2016;21:1608-1612.
6. Lambert JC, Heath S, Even G, et al. Genome-wide association study identifies variants at CLU and CR1 associated with Alzheimer's disease. Nat Genet 2009;41:1094-1099.
7. Jun G, Ibrahim-Verbaas CA, Vronskaya M, et al. A novel Alzheimer disease locus located near the gene encoding tau protein. Molecular psychiatry 2016;21:108-117.
8. Jun GR, Chung J, Mez J, et al. Transethnic genome-wide scan identifies novel Alzheimer's disease loci. Alzheimers Dement 2017;13:727-738.
9. Jiang Q, Jin S, Jiang Y, et al. Alzheimer’s Disease Variants with the Genome-Wide Significance are Significantly Enriched in Immune Pathways and Active in Immune Cells. Molecular neurobiology 2017;54:594-600.
10. Kunkle BW, Grenier-Boley B, Sims R, et al. Genetic meta-analysis of diagnosed Alzheimer’s disease identifies new risk loci and implicates Aβ, tau, immunity and lipid processing. Nature genetics 2019;51:414-430.
